# Extracellular Vesicles Generated by Gram-Positive Bacteria Protect Human Tissues *Ex Vivo* From HIV-1 Infection

**DOI:** 10.3389/fcimb.2021.822882

**Published:** 2022-01-25

**Authors:** Paolo E. Costantini, Christophe Vanpouille, Andrea Firrincieli, Martina Cappelletti, Leonid Margolis, Rogers A. Ñahui Palomino

**Affiliations:** ^1^Section of Intercellular Interaction, Eunice Kennedy Shriver National Institute of Child Health and Human Development, National Institutes of Health, Bethesda, MD, United States; ^2^Department of Pharmacy and Biotechnology, University of Bologna, Bologna, Italy

**Keywords:** vaginal microbiota, gram positive bacteria, HIV-1, extracellular vesicles (EVs), *Gardnerella vaginalis*, *Staphylocccus aureus*, *Enteroccoccus faecalis*, *Enteroccoccus faecium*

## Abstract

Vaginal microbiota dominated by lactobacilli protects women from sexually transmitted infection, in particular HIV-1. This protection is, in part, mediated by *Lactobacillus*-released extracellular vesicles (EVs). Here, we investigated whether EVs derived from other Gram-positive bacteria also present in healthy vaginas, in particular *Staphylococcus aureus*, *Gardnerella vaginalis*, *Enterococcus faecium*, and *Enterococcus faecalis*, can affect vaginal HIV-1 infection. We found that EVs released by these bacteria protect human cervico-vaginal tissues *ex vivo* and isolated cells from HIV-1 infection by inhibiting HIV-1-cell receptor interactions. This inhibition was associated with a diminished exposure of viral Env by steric hindrance of gp120 or gp120 modification evidenced by the failure of EV-treated virions to bind to nanoparticle-coupled anti-Env antibodies. Furthermore, we found that protein components associated with EV’s outer surface are critical for EV-mediated protection from HIV-1 infection since treatment of bacteria-released EVs with proteinase K abolished their anti-HIV-1 effect. We identified numerous EV-associated proteins that may be involved in this protection. The identification of EVs with specific proteins that suppress HIV-1 may lead to the development of novel strategies for the prevention of HIV-1 transmission.

## Introduction

*Lactobacillus* spp. in the vaginal niche of premenopausal women, when dominant, protect from several gynaecological infections (O’Hanlon et al., 2013; Parolin et al., 2015; Nardini et al., 2016; Gosmann et al., 2017; Siroli et al., 2017; Parolin et al., 2018). Indeed, a shift from *Lactobacillus* dominance to non-*Lactobacillu*s dominance increases the colonization and/or overgrowth of pathogenic or opportunistic bacteria such as *Gardnerella* spp., *Prevotella*, and *Clostridium*, which are often found during bacterial vaginosis (Fredricks and Marrazzo, 2005; Fredricks et al., 2005; Oakley et al., 2008; Srinivasan and Fredricks, 2008; Ravel et al., 2013). Like bacterial vaginosis, aerobic vaginitis is featured by a marked rearrangement in the microbial community. In this case, the decrease in abundance of lactobacilli is associated with the overgrowth of aerobic bacteria mainly belonging to the genera *Staphylococcus*, *Streptococcus*, *Escherichia*, and *Enterococcus* (Donders, 2007). Nevertheless, in small amounts, these opportunistic/pathogenic bacteria can be present in healthy vaginal microbiota (Ravel et al., 2011).

Numerous studies have reported that vaginal lactobacilli protect from HIV-1 infection through acidification of the vaginal niche, stimulation of an anti-HIV-1 immune response, and capture of HIV-1 virions by membrane lectins (Atashili et al., 2008; Petrova et al., 2013; Gosmann et al., 2017; Ñahui Palomino et al., 2017). Recently, another mode of lactobacilli-mediated protection against HIV-1 infection was discovered, that is the release of extracellular vesicles (EVs) (Ñahui Palomino et al., 2019).

EVs are considered to be important mediators for cell–cell communications and constitute nanosized proteolipid particles carrying numerous bioactive molecules and covered by a lipid bilayer membrane (Nagakubo et al., 2019; Ñahui Palomino et al., 2021). The complexity of EVs reflects their multifunctionality, since EVs can shuttle numerous bioactive molecules that are involved during bacterial–bacterial interactions such as antibiotic resistance, stress resistance, horizontal gene transfer, and competition with other microorganisms; bacterial EVs are also involved during bacterial–host interactions, such as immune modulation and delivery of virulence factor, among others (Mashburn and Whiteley, 2005; Oakley et al., 2008; Manning and Kuehn, 2011; Kim et al., 2015; Stubbendieck and Straight, 2016; Domingues et al., 2017; Ñahui Palomino et al., 2021). Despite several functions described for bacterial EVs in the last decade, little is known about their possible interactions with viruses. In our previous work, we reported on the HIV-1 inhibition by *Lactobacillus*-derived EVs (*L. crispatus* BC3 and *L. gasseri* BC12) in both human cell lines and *ex-vivo* tissues (Ñahui Palomino et al., 2019). This inhibition was associated with the binding of *Lactobacillus*-derived EVs to HIV-1 Env protein, resulting in a reduction of HIV-1 entry and binding to target cells (Ñahui Palomino et al., 2019).

Here, we investigated whether EVs derived from four opportunistic human pathogenic bacteria (*S. aureus*, *E. faecium*, *E. faecalis* and *G. vaginalis*) affect HIV-1 infection of human cell cultures *in vitro* as well of human cervico-vaginal tissues *ex-vivo*. *Ex vivo* tissue cultures allow to maintain the complex cytoarchitecture of the tissue and thus constitute an adequate experimental model to study HIV-1 pathogenesis (Grivel and Margolis, 2009).

Our results demonstrate that bacterial EVs derived from Gram-positive (*S. aureus*, *E. faecium*, *E. faecalis*) and Gram-variable (*G. vaginalis*) bacteria inhibit HIV-1 infection of human lymphoid T-cell line and human cervico-vaginal tissues *ex vivo*. This protection is exerted by the protein components exposed on the EV surface and is associated, at least in part, with obstruction of the gp120 protein of the viral envelope.

## Materials and Methods

### Bacterial Strains and Extracellular Vesicle Isolation and Characterization

In this study, we used the Gram-variable bacteria *Gardnerella vaginalis* ATCC14018 and the following Gram-positive bacteria: *Staphylococcus aureus* ATCC12600, *Enterococcus faecium* ATCC19434, and *Enterococcus faecali*s ATCC19433. All bacterial strains were cultured at 37°C in de Man, Rogosa, and Sharpe (MRS) broth (Difco, Detroit, MI) pre-filtered with 0.1-μm filters to decrease the number of particles that are present in the medium. *G. vaginalis* was cultured in anaerobic conditions in jars containing Gaspak EZ (BD, Franklin Lakes, NJ), while the remaining strains (*S. aureus, E. faecium*, and *E. faecalis*) were cultured in aerobic conditions. The optical density (OD) of overnight cultures were measured using the spectrophotometer (Biophotometer, Eppendorf, Germany) and the bacterial concentration was calculated using an OD_600_ nm conversion factor of 0.4, corresponding to a concentration of 10^8^ colony-forming units (CFU)/mL. Bacterial EVs were isolated from the supernatants of bacterial cultures by ultracentrifugation (Théry et al., 2006; Ñahui Palomino et al., 2019). Briefly, tubes containing 50 mL of bacterial cultures were centrifuged at 2,800 × *g* for 15 min at 4°C. The supernatants obtained were filtered with 0.22-μm filters to remove any remaining bacteria. Then, bacterial EVs were isolated by ultracentrifugation at 100,000 × *g* for 70 min at 4°C (Ultracentrifuge WX ultra 80, Thermo Fisher Scientific). EV-free supernatant was removed and collected for further experiments, while the pellet, containing bacterial EVs, was resuspended in 150 μL of PBS and stored at 4°C. A similar approach was used to isolate EVs from MRS medium.

Bacterial EVs were characterized in terms of size and concentration through nanoparticle tracking analysis (NTA) using the NanoSight NS300 (Malvern instruments Ltd, Malvern, UK) equipped with a 405-nm laser. The isolated bacterial EVs were diluted 1:100 in PBS and loaded in 1-mL syringes placed in a syringe pump controller. The samples were analyzed for 60 seconds three times with camera level = 13, detect threshold = 6, and syringe pump speed = 15. NTA results were analyzed using NTA software, version 3.1.54 (Malvern instruments Ltd, Malvern, UK).

### Cell and Human Cervico-Vaginal Tissue Cultures

Human T-lymphocytes MT-4 (NIH AIDS Reagent Program, Germatown, MD, catalog number 120) were cultured in Roswell Park Memorial Institute (RPMI) 1640 medium (Gibco BRL, Carlsbad, CA) supplemented with 10% heat-inactivated fetal bovine serum (FBS).

Human cervico-vaginal tissues were received as anonymized samples, obtained from routine hysterectomies through the National Disease Research Interchange (NDRI, Philadelphia, PA). The protocol was approved by the NDRI IRB#5 of the University of Pennsylvania. NDRI maintains a Federal Wide Assurance (FWA00006180) agreement with the DHHS, Office for Human Research Protections to comply with federal regulations concerning research involving human subjects. Tissues were dissected as described previously (Grivel and Margolis, 2009) with slight modifications. Briefly, the mucosa layers from ecto- and endo-cervix tissues were cut in blocks of 2 mm^3^, and blocks were placed onto collagen sponge gel (Gelfoam, Pfizer, New York, NY) at the air–liquid interface and cultured in RPMI 1640 medium supplemented with FBS at 15%, 1 mM non-essential amino acids, 1 mM sodium pyruvate, amphotericin B at 2.5-μg/mL, and gentamycin sulfate at 50-μg/mL.

### Anti-HIV-1 Assays

Anti-HIV-1 properties of bacterial supernatants and bacterial-derived EVs were evaluated in human T-lymphocyte MT-4 cell line.

Anti-HIV-1 effect was tested in MT-4 cells infected with a prototypic X4 HIV-1 isolate, LAI.04 (HIV-1_LAI.04_; Rush University Virology Quality Assurance Laboratory, Chicago, IL) as described earlier (Ñahui Palomino et al., 2019). Briefly, 30 µL of HIV-1_LAI.04_ viral stock (350 ng p24_gag_/mL) was treated with 1×10^10^ bacterial EVs (derived from *S. aureus*, *E. faecium*, *E. faecalis*, or *G. vaginalis*), or with bacterial supernatants (*S. aureus*, *E. faecium*, *E. faecalis*, *G. vaginalis*) at 0.5%, or with bacterial EV-free supernatants at 0.5% (after ultracentrifugation, *S. aureus*, *E. faecium*, *E. faecalis*, *G. vaginalis*), or with 1×10^10^ particles isolated from MRS medium, or controls (PBS, MRS medium), for 1 h, at 37°C. EV/HIV-1_LAI.04_ or EV-free/HIV-1_LAI.04_ mixtures were then used to infect 3×10^5^ MT-4 cells for 1 h at 37°C under constant agitation at 400 rpm. Following infection, cells were washed three times with 1 mL of PBS and centrifuged for 5 min at 400 g to eliminate free virions. Infected cells were resuspended in 3 mL of RPMI medium containing the corresponding 1×10^10^ EVs/mL, or PBS, or MRS medium. Cells were transferred to 24-well plates (1 mL/well, 1×10^5^ cells/well; Sigma-Aldrich, St. Louis, MO) and incubated for 3 days at 37°C. Experiments of the same type were conducted to address the concentration-dependent antiviral activity of bacterial EVs derived from *S. aureus*, *E. faecium*, *E. faecalis*, and *G. vaginalis*, using 1×10^7^, 1×10^8^, 1×10^9^, or 1×10^10^ bacterial EVs/mL.

To digest the EV protein surface, 150 µL of bacterial EVs (stock 1×10^10^ EVs/mL) were treated with proteinase K (PK) at a final concentration of 0.2 mg/mL (ThermoFisher Scientific, Waltham, MA) for 1 h. PK-treated EVs were resuspended with PBS to a final volume of 30 mL. Then, bacterial EVs were isolated by ultracentrifugation at 100,000 × *g* for 70 min at 4°C (Ultracentrifuge WX ultra 80, Thermo Fisher Scientific). The pellet, containing bacterial EVs, was resuspended in 150 μL of PBS. In control experiments, PK alone was used. The anti-HIV-1 effect of EVs previously treated with PK (1×10^10^ EVs/mL derived from *S. aureus*, *E. faecium*, *E. faecalis*, or *G. vaginalis*) was evaluated in MT-4 cells as described above.

To study whether bacterial EVs induce anti-HIV-1 responses in host cells, 3×10^5^ MT-4 cells were cultured in cell media containing or not containing 1×10^10^ bacterial EVs derived from *S. aureus*, *E. faecium*, *E. faecalis*, or *G. vaginalis*, for 24 h at 37°C. Then, cells were washed three times with 1 mL of PBS and centrifuged for 5 min at 400 g to eliminate bacterial EVs. Thirty µL of HIV-1_LAI.04_ (stock 350 ng p24_gag_/mL) was then used to infect MT-4 cells for 1 h at 37°C under constant agitation at 400 rpm. After infection, cells were washed three times with 1 mL of PBS and centrifuged for 5 min at 400 g to eliminate free virions. Infected cells were resuspended in 3 mL of RPMI medium, transferred at 1 mL/well to a 24-well plate, and incubated for 3 days at 37°C.

Also, the anti-HIV-1 effect of bacterial EVs was evaluated in human cervico-vaginal tissues *ex vivo*. To infect human cervico-vaginal tissues *ex vivo*, tissue blocks were infected with the prototypic R5 HIV-1 isolate BaL (HIV-1_BaL_; Rush University Virology Quality Assurance Laboratory). HIV-1_BaL_ viral stock (400 µL, with 120 ng p24_gag_/mL) was preincubated with 1×10^10^ bacterial EVs (derived from *S. aureus*, *E. faecium*, *E. faecalis*, or *G. vaginalis*), or with 1×10^10^ particles isolated from MRS medium, or with PBS only (control), for 1 h at 37°C. Then, cervico-vaginal tissues were infected with an EV/HIV-1_BaL_ or EV-free/HIV-1_BaL_ mixture for 2 h at 37°C in agitation. Afterward, the infected cervico-vaginal tissue blocks were washed three times with PBS and transferred at the liquid–air interface onto Gelfoam (nine blocks/well) containing 1 mL of RPMI medium supplemented or not with 1×10^10^ bacterial EVs/mL. The tissue blocks were kept for 12 days at 37°C, replacing and collecting the medium containing or not containing bacterial EVs (1×10^10^ EVs/mL) every 3 days.

### Measurement of HIV-1 Replication

In all the experiments, we evaluated HIV-1 replication by measuring the HIV-1 capsid protein, p24gag antigen, released in cell or tissue culture medium, using an immunofluorescent cytometric bead-based assay by Luminex (Biancotto et al., 2009). Briefly, 15 µL/well of each sample were initially transferred to a 96-well plate and lysed with 135 µL/well of Luminex lysis buffer (PBS containing 1% Triton X-100, 0.02% Tween20, 0.02% BSA, and 20 mM Tris-HCl pH 6) for 30 min at 37°C. The lysed samples were mixed 1:1 (50 µL:50 µL) with a solution containing p24 antibodies coupled to magnetic beads at a concentration of 1×10^3^ beads/mL and incubated for 1 h at room temperature, in agitation at 400 rpm. Magnetic beads were previously coupled with anti-p24 antibody. Then, each plate was washed twice with 200 µL of Luminex wash buffer (PBS containing 0.02% Tween20, 20 mM Tris-HCl pH 6) using an ELx405 magnetic microplate washer (BioTek, Winooski, VT); then 100 µL of the detection antibody RD1-anti-p24 (Beckman Coulter, Indianapolis, IN) was added to each well (final concentration 0.5 μg/mL), followed by 1 h of incubation at room temperature, in agitation at 400 rpm. The plates were washed twice, as described above. To measure the p24 concentration, 100 µL/well of Luminex buffer was added (PBS containing 0.02% Tween20, 0.02% BSA, 20 mM Tris-HCl pH 6). The latter was performed on a Luminex 200 (BioRad, Hercules, CA) using its Bioplex manager software version 6.0.

### Cell Viability

We performed cell viability assays in MT-4 cells using the automated cell counting system Cellometer Auto 2000 (Nexcelom bioscience, San Diego, CA). We determined the numbers of total and dead cells in control cultures, in bacterial EV-treated cultures, and in bacterial supernatant cultures, staining the cells with a dual-fluorescence acridine orange/propidium iodide (AOPI, Nexcelom bioscience, San Diego, CA). Acridine Orange stains all nucleated cells to generate green fluorescence. Propidium iodide stains all dead nucleated cells to generate red fluorescence. Briefly, cells were seeded to 24-well plates at a final concentration of 1×10^5^ cells per well and treated with 1×10^10^ EVs/mL, or bacterial supernatants at 0.5%, or PBS. After day 3 of cell culture, 20 µL of cell suspensions was mixed with 20 µL of AOPI staining solution. Cell viability was expressed as percentage of viable cells in EV-free or EV-treated cells or in bacterial supernatants treated cells.

### HIV-1 Capture Assays

HIV-1 capture experiments were performed to study the interactions of bacterial-derived EVs (*S. aureus*, *E. faecium*, *E. faecalis*, and *G. vaginalis*) with the HIV-1 envelope proteins gp120 and gp41.

Human monoclonal PG9 antibody (stock 1 mg/mL; Polymun Scientific, Austria) was used to capture HIV-1 by binding the trimeric form of viral gp120. Human monoclonal VRC01 antibody (stock 1 mg/mL; NIH AIDS Reagent Program, Germatown, MD) was used to capture HIV-1 through its CD4 binding site on gp120. Human monoclonal 4B3 antibody (stock 1 mg/mL; Polymun Scientific, Austria) was used to capture HIV-1 virions with gp41. The antibodies were previously coupled to 15-nm carboxyl-terminated magnetic iron oxide nanoparticles (MNPs) according to manufacturer’s protocol (Ocean NanoTech, San diego, CA). Towards this goal, 50 µL of HIV-1_LAI.04_ was incubated with 50 µL of bacterial EVs at a concentration of 1×10^10^ particles/mL or with PBS (control) for 1 h at 37°C under constant agitation at 400 rpm. Thereafter, viral particles were captured by adding 50 µL of MNPs coupled to monoclonal antibody PG9, VRC01, or 4B3 and incubated for 1 h at 37°C, under constant agitation at 400 rpm. To evaluate the number of viruses captured, the virions captured with PG9, VRC01, or 4B3 MNPs were separated from free virions using magnetic columns inserted in a high field MACS magnet (Miltenyi Biotech, Auburn, CA). Columns were then washed 3 times with 600 µL of washing buffer (0.5% BSA, 1 mM EDTA), demagnetized for 5 min, and eluted with 600 µL of Luminex lysis buffer (PBS containing 1% Triton X-100). The p24gag antigen concentration in the eluate was measured as described above. Experiments of a similar type were performed to capture HIV-1 virions with PG9-MNPs or VRC01-MNPs after exposure of HIV-1 to PK-treated bacterial EVs (1×10^10^ EVs).

### Proteomic Analysis

Three independent replicates from EVs derived from *S. aureus*, *E. faecium*, *E. faecalis*, and *G. vaginalis* were analyzed for proteomics with mass spectroscopy (BGI Americas, San Jose, CA) as follows:

**Sample lysis:** About 100 µL of bacterial EVs were mixed with 700 µL of lysis buffer (9M urea, 0.5% Waters Rapigest, pH 8.5). Samples were waterbath-sonicated for 30 min followed by centrifugation for 10 min at 14,000 *g*. Protein concentration of lysates were measured by BCA assay (Cat No: A53225, Thermo Fisher Scientific).

**Proteomics sample preparation:** About 20 µg of each sample was taken from the lysate and normalized to the same volume with lysis buffer. Samples were reduced in 10 mM dichlorodiphenyltrichloroethane (DTT) for 25 min at 60°C, and then reduced samples were alkylated in 20 mM iodoacetamide (IAM) in a dark environment for 20 min at room temperature. Excess IAM in the samples were quenched by adding 100 mM DTT solutions. Deionized water and HEPES pH 8.5 were added to each sample, so that final urea concentration was diluted to 1.6 M, and the final pH was about 8 for enzymatic digestion. Try/LysC (1 µg) (Cat No: A41007, Thermo Fisher Scientific) was added to each sample. Samples were incubated overnight at 37°C for 12 h. An additional 1 µg of Tryp/LysC was added to each sample the next day, and samples were incubated for 4 h to complete the enzymatic digestion; 10% trifluoroacetic acid (TFA) was added into each digested peptide sample to a final concentration of 1% TFA. Then, the acidified samples were desalted using C18 stage tips (Cat # PTR-92-05-18, Biotage), and the samples were ready for LC/MS analysis.

**LC/MS analysis:** All samples were analyzed by using nano flow HPLC (Ultimate 3000, Thermo Fisher Scientific) followed by Thermo Orbitrap Mass Spectrometer (Q-Exactive HF-X). Nanospray Flex™ Ion Source (Thermo Fisher Scientific) was equipped with Column Oven (PRSO-V2, Sonation) to heat up the nano column (PicoFrit, 100 µm x 250 mm x 15 µm tip, New Objective) for peptide separation. The nano LC method is water acetonitrile-based, lasts 150 minutes with a 0.25-µL/min flowrate. For each LC/MS run, all peptides were first engaged on a trap column (Cat. No: 160454, Thermo Fisher) and then were delivered to the separation nano column at the mobile phase. A protein profiling specific data dependent acquisition– (DDA) based mass spectrometry method on QE HF-X was used to sequence digested peptides that were eluted from the nano column. For the full MS, a 60,000 resolution was used with 3E6 AGC target or 50ms IT, and the scan range was 400 m/z–1,600 m/z. For the dd-MS2(MS/MS), a 15,000 resolution was used with 1E5 AGC target or 40ms IT. The isolation window was 1.4 Da. Normalized Collision Energy (NCE) was set to 27 with a 10-cycle loop.

**Bioinformatic analysis pipeline overview:** Collected LC-MS data were analyzed by Proteome Discoverer 2.5 (Thermo Fisher Scientific). Area under the curve (AUC) quantitative proteomics searches were performed. All searches were performed in Sequest HT node with mass tolerance of 20 ppm MS1 and 0.05 Da for MS2. Multiple databases (UP000310049, UP000005269, UP000001415, UP000033074, UP000002371, UP000030761) from Swiss-Prot were used to search the corresponding species of exosomes. Perculator node was used for peptide FDR filtering (Strict: 001, relaxed: 0.05). Peptide abundances were normalized by total peptide abundance and quantified by MS1 level AUC.

**Filtering and identification of orthologue proteins in *S. aureus*, *G. vaginalis*, *E. faecium*, and *E. faecalis* EV proteomes:** For each strain, the EV-derived proteomic data were initially filtered out to remove proteins that i) were identified as contaminants, i.e. of human origin, ii) were not detected in all the replicates. Redundant proteins were also excluded by keeping only the BLAST Reciprocal Best Hits (RBH) (https://github.com/peterjc/galaxy_blast/tree/master/tools/blast_rbh) that were identified from comparisons of each EV-derived proteome (predicted using the Proteome Discover UniProt database) with the corresponding NCBI RefSeq strain-specific proteome (*S. aureus*: GCF_001879295.1, *G. vaginalis*: GCF_004336685.1, *E. faecium*: GCF_900447735.1, *E. faecalis*: GCF_000392875.1). These resulting curated EV-derived proteomes were further analyzed using OrthoFinder (Emms and Kelly, 2019) to identify orthologue proteins shared among the four strains.

**Gene Ontology annotation functional enrichment:** Gene ontology (GO) annotation was performed using eggNOG-mapper v2 against the database eggNOG v5.0 (Cantalapiedra et al., 2021). The R package topGO was used to identify the molecular function GO terms that were enriched in the shared EVs orthologue dataset (test set) as compared with each strain EV-derived proteome (universe set). Significantly enriched GO terms were detected by application of the ‘weight01’ algorithm and the Fisher test implemented in topGO (Alexa and Rahnenfuhrer, 2019). TopGO results were finally manually curated to remove redundant GO terms including the same set of proteins.

**Identification of cytoplasmic, membrane, and extra-cytoplasmic proteins:** TMHMM v. 2.0 was used in combination with SignalP v5.0 to distinguish cytoplasmic, membrane and extracytoplasmic proteins. Proteins without trans-membrane (TM) domains but with a signal peptide were identified as putatively extracytoplasmic, which could correspond to either periplasmic or extracellular proteins. Conversely, proteins with TM motifs and a signal peptide were marked as membrane.

## Results

### Gram-Positive Bacteria Secrete EVs

**Figure 1:**
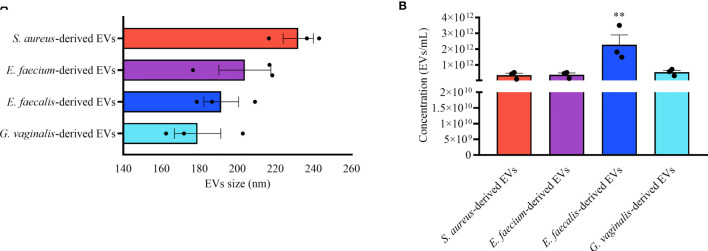
Bacterial EV-size and concentration. Nanoparticle tracking analysis of EVs derived from *S. aureus, E. faecium, E. faecalis*, and *G. vaginalis*. **(A)** EV-sizes expressed as mean ± SEM of particle diameter (nm). **(B)** Mean ± SEM of the EV concentration (particles/mL). Presented are the results of three independent measurements. **p < 0.01.

EVs were isolated from the following four opportunistic human pathogens: *Staphylococcus aureus* ATCC12600, *Enterococcus faecium* ATCC19434, *Enterococcus faecalis* ATCC19433, and *Gardnerella vaginalis* ATCC14018. EVs were isolated from bacterial cultures by ultracentrifugation followed by its characterization in terms of size and concentration using nanoparticle tracking analysis (NTA). Our results demonstrated that all bacterial strains secrete EVs of similar size ranging from 179.07 ± 17.22 nm (*G. vaginalis*) to 231.97 ± 11.24 nm (*S. aureus*) (**Figure 1A**). *E. faecali*s released EVs of around one order of magnitude higher than the other strains, 2.27 ± 0.88×10^12^ particles/mL (**Figure 1B**). *S. aureus* released 3.52 ± 1.79×10^11^, *E. faecium* released 3.78 ± 1.61×10^11^, and *G. vaginalis* released 5.42 ± 1.65×10^11^ particles/mL (**Figure 1B**).

### Gram-Positive–Derived EVs Suppress HIV-1 Infection in Human MT-4 Cells

**Figure 2:**
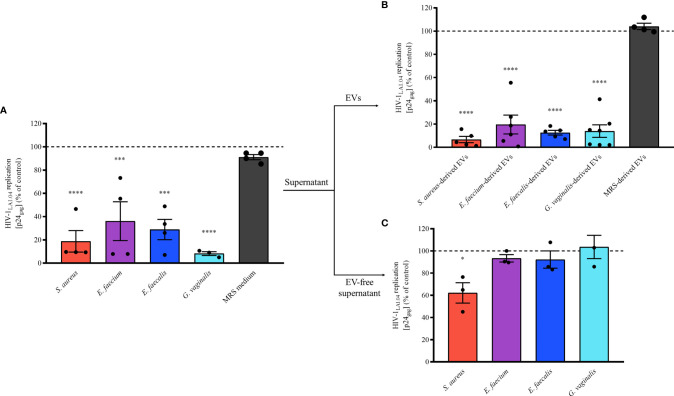
Antiviral effect of bacterial-derived supernatants, EVs, and EV-free supernatants. A mixture of HIV-1_LAI.04_ and bacterial-derived supernatants (0.5%), EVs (1×10^10^ EVs/mL), or EV-free supernatants (0.5%) incubated for 1 h was added to MT-4 cell cultures. Cells were washed and cultured for 3 days in the presence of bacterial supernatants, EVs, or EV-free supernatants. In control experiments, EV-free, particles isolated from fresh MRS medium, and MRS medium were tested as well. Replication of HIV-1 was evaluated from measurements of the capsid protein p24_gag_, in the cell culture medium; data are presented as percentages of HIV-1 replication compared with untreated controls. **(A)** The effects of bacterial supernatants (0.5%) derived from *S. aureus*, *E. faecium*, *E. faecalis, G. vaginalis*, and MRS medium (0.5%) on HIV-1 replication in MT-4 cells. **(B)** The effects of 1×10^10^ EVs/mL bacterial EVs derived from *S. aureus*, *E. faecium*, *E. faecalis, G. vaginalis*, and MRS medium-derived particles on HIV-1 replication in MT-4 cells. **(C)** The effects of bacterial EV-free supernatants (0.5%, after pulling down EVs by ultracentrifugation) derived from *S. aureus*, *E. faecium*, *E. faecalis*, and *G. vaginalis* on HIV-1 replication in MT-4 cells. Presented are means ± SEM from at least three independent measurements. Asterisks indicate statistical significance by one-way ANOVA multiple comparison with Dunnett’s correction (*p < 0.05, ***p < 0.001, ****p < 0.0001).

To investigate whether Gram-positive bacteria secrete anti-HIV-1 compounds, we tested the anti-HIV-1 activity of the supernatants derived from overnight cultures of the four pathogenic bacteria on HIV-1 replication in human lymphoid MT-4 cells. MT-4 cells infected with HIV-1_LAI.04_ were cultured in cell culture medium supplemented with bacterial supernatants at 0.5% (diluted 1:200 in cell culture medium). We found that HIV-1_LAI.04_ replication was reduced by 81.26 ± 18.52% (*S. aureus*, *p* < 0.0001, *n* = 4), 63.93 ± 33.40% (*E. faecium*, *p* = 0.0004, *n* = 4), 71.14 ± 17.40% (*E. faecalis*, *p* = 0.0001, *n* = 4), and 91.77 ± 2.85% (*G. vaginalis*, *p* < 0.0001, *n* = 3) (**Figure 2A**) compared with controls. Also, there was no inhibition of HIV-1 in MT-4 cells treated with MRS, the medium used to grow bacteria (8.91 ± 4.43%, *p* = 0.9272, *n* = 4).

These data show that *S. aureus, E. faecium, E. faecalis*, and *G. vaginalis* secrete antiviral factors extracellularly. On the basis of previous findings (Ñahui Palomino et al., 2019), we then isolated EVs from bacterial supernatants and tested their effects on HIV-1 infection in MT-4 cells. Our results showed that in the presence of any of the pathogen-derived EVs, HIV-1_LAI.04_ replication was significantly reduced compared with the control (**Figure 2B**). In particular, HIV-1_LAI.04_ replication was reduced when MT-4 cells were treated with 1 x 10^10^ bacterial EVs by 93.28 ± 5.38% (*S. aureus*, *p* < 0.0001, *n* = 5), 80.33 ± 18.09% (*E. faecium*, *p* < 0.0001, *n* = 6), 87.37 ± 3.97% (*E. faecalis*, *p* < 0.0001, *n* = 5), and 86.02 ± 13.13% (*G. vaginalis*, *p* < 0.0001, *n* = 7). On the contrary, as expected, particles isolated from MRS medium did not reduce HIV-1 replication (*p* = 0.9723, *n* = 4).

To determine whether antiviral factors other than bacterial EVs were present in the bacterial supernatants, we tested the effects of bacterial EV-depleted supernatants (0.5% of EV-free supernatants) on HIV-1 replication in MT-4 cells. As shown in **Figure 2C**, the depletion of EVs from bacterial supernatants caused a significant loss of their anti-HIV activities. Indeed, HIV-1 replication in MT-4 cells cultured in EV-free supernatant were similar to control experiments (100%), at 93.32 ± 5.80% (*E. faecium*, *p* = 0.9115, *n* = 3), 92.24 ± 13.52% (*E. faecalis*, *p* = 0.8628, *n* = 3), and 92.24 ± 18.14% (*G. vaginalis*, *p* = 0.9893, *n* = 3). Nevertheless, EV-free supernatant derived from *S. aureus* still significantly decreased HIV-1 replication by 37.82 ± 15.86% (*p* = 0.0141, *n* = 3), suggesting the presence of other antiviral factors in the supernatant of *S. aureus*.

**Figure 3:**
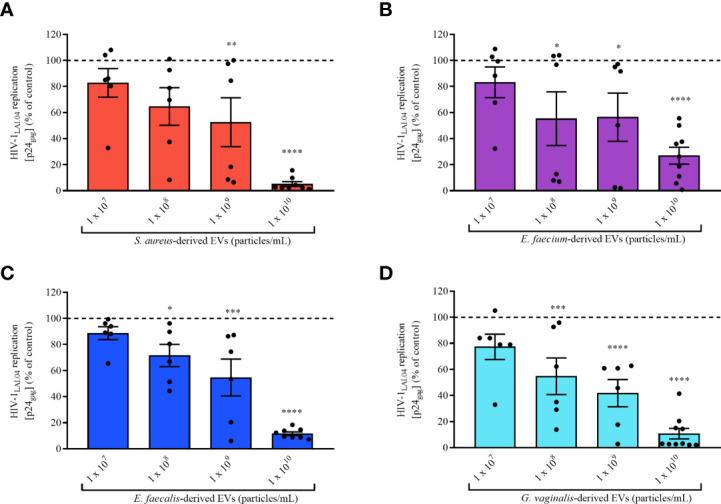
Concentration effect of bacterial EVs on HIV-1 replication. MT-4 cells infected with HIV-1_Lai.04_ were cultured in presence of bacterial EVs [*S. aureus* **(A)**, *E. faecium* **(B)**, *E. faecalis* **(C)**, and *G. vaginalis* **(D)**] at different EV concentrations, 1×10^7^, 1×10^8^, 1×10^9^, and 1×10^10^ EVs/mL. After 3 days of culture, HIV-1 replication was evaluated from measurements of the concentration of p24_gag_ in the cell culture medium. The data are presented as percentages of HIV-1 replication compared with untreated controls. Presented are means ± SEM from six independent measurements. Asterisks indicate statistical significance by one-way ANOVA multiple comparison with Dunnett’s correction (*p < 0.05, **p < 0.01, ***p < 0.001, ****p < 0.0001).

Next, we assessed the concentration-dependent anti-HIV-1 effect of bacterial EVs by testing different EV concentrations, 1×10^7^, 1×10^8^, 1×10^9^, and 1×10^10^ EVs/mL. As shown in **Figure 3**, the anti-HIV-1 effect exerted by bacterial EVs from each bacterial species was in a concentration dependent manner. HIV-1 replication in MT-4 cells was reduced by 47.45 ± 45.89% when incubated with *S. aureus*-derived EVs starting at 1×10^9^ EVs/mL (**Figure 3A**, *p* = 0.0094, *n* = 6), or by 44.69 ± 50.45% by *E. faecium*-derived EVs starting at 1×10^8^ EVs/mL (**Figure 3B**, *p* = 0.0378, *n* = 6), or by 28.51 ± 20.92% by *E. faecalis*-derived EVs starting at 1×10^8^ EVs/mL (**Figure 3C**, *p* = 0.0199, *n* = 6), or by 45.24 ± 34.35% by *G. vaginalis*-derived EVs starting at 1×10^8^ EVs/mL (**Figure 3D**, *p* = 0.0005, *n* = 6). On the other hand, decreasing the amount of bacterial EVs below 1×10^7^ EVs/mL led to a loss of anti-HIV-1 activity, demonstrating that the antiviral effect of pathogen-derived EVs is dose-dependent (**Figure 3**).

### EV Suppression of HIV-1 Replication Is Not Due to Cell Cytotoxicity

**Figure 4:**
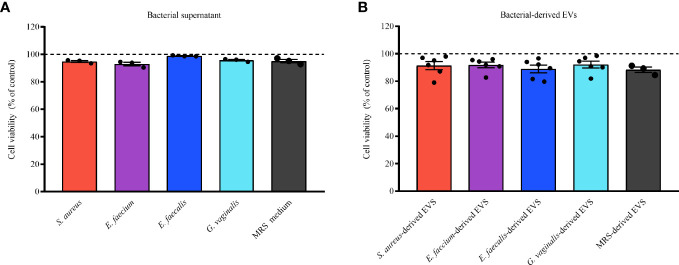
Cell viability of cells treated with bacterial supernatants or EVs. MT-4 cells were treated or not treated for 3 days with bacterial supernatants (0.5%) or EVs (1×10^10^ EVs/mL) derived from *S. aureus*, *E. faecium*, *E. faecalis*, and *G. vaginalis*. MRS medium or particles derived from MRS were used as controls. The numbers of viable and dead cells were counted according to orange acridine/propidium-iodide-based assay. Results are expressed as percentages of cell viability in EV-free or EV-treated cells. Presented are means ± SEM from five independent measurements. Cell viability in presence of bacterial supernatants **(A)** or EVs **(B)** derived from *S. aureus*, *E. aecium*, *E. faecalis*, and *G. vaginalis* is shown. Results are expressed as percentages of viable cells treated or not treated with bacterial supernatant or EVs. Presented are means ± SEM from at least three independent measurements.

To exclude the possibility that the bacterial EV- or bacterial supernatant- anti-HIV-1 effects were due to cell cytotoxicity, we evaluated the possible cytotoxic effect of bacterial supernatants (diluted 1:200 in cell culture medium) and EVs in MT-4 cells. As shown in **Figure 4**, there was no decrease in cell viability after treatment with bacterial supernatants (**Figure 4A**) or bacterial EVs (**Figure 4B**) in comparison with untreated control. All the bacterial supernatants tested, as well as MRS medium, were not cytotoxic for MT-4 cells. Indeed, cell viability treated with bacterial supernatants was statistically not different from that in control experiments (100%), ranging from 92.93 ± 2.21% (*E. faecium*, *p* = 0.8841, *n* = 3) to 98.87 ± 0.38% (*E. faecalis*, p = 0.0107, *n* = 3) (**Figure 4A**). Similarly, bacterial EV-treatment of MT-4 cells did not alter cell viability compared with controls, ranging from 88.93 ± 6.85% (*E. faecalis*-derived EVs, *p* = 0.8603, *n* = 6) to 92.13 ± 6.01% (*G. vaginalis*-derived EVs, *p* = 0.9999, *n* = 6) (**Figure 4B**).

### Gram-Positive-Derived EVs Suppress HIV-1 Infection in Human Cervico-Vaginal Tissues *Ex Vivo*

**Figure 5:**
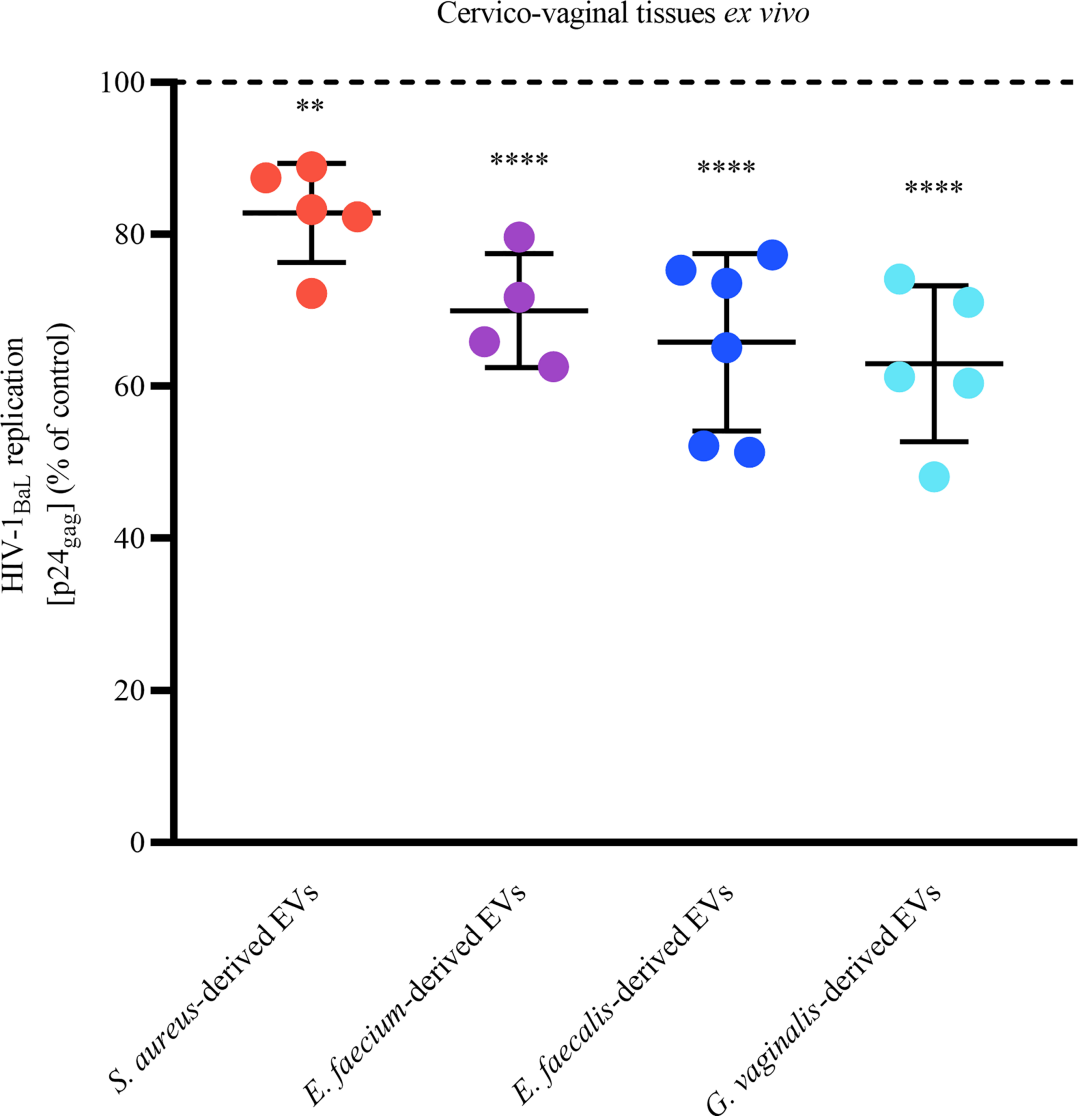
Anti-HIV-1 effect of bacterial EVs in human cervico-vaginal tissues *ex vivo*. Cervico-vaginal tissue blocks were infected with EV-pretreated HIV-1_BaL_ and cultured for 12 days, with replacement every 3 days of tissue culture medium containing or not containing 1×10^10^ EVs/mL derived from *S. aureus*, *E. faecium*, *E. faecalis*, and *G. vaginalis*. Replication of HIV-1 was evaluated from measurements of the capsid protein p24_gag_ in tissue culture medium and is represented as a percentage of HIV-1 replication in untreated control. Presented are means ± SEM from tissues of at least four donors. Asterisks indicate statistical significance by one-way ANOVA multiple comparison with Dunnett’s correction (**p < 0.01, ****p < 0.0001).

On the basis of the EVs’anti-HIV-1 effect observed in MT-4 cells, we tested the effect of bacterial EVs against HIV-1 replication in an *in vivo*–like system of human cervico-vaginal tissues *ex vivo*. Toward this goal, human cervico-vaginal tissues infected with HIV-1_BaL_ were cultured in medium containing or not containing bacterial EVs (1x10^10^ EVs/mL). We found that EVs derived from all bacteria significantly reduced HIV-1 replication (**Figure 5**). In particular, *S. aureus*-derived EVs decreased HIV-1 replication by 17.17 ± 6.52% (*p* = 0.009, *n* = 5), *E. faecium*-derived EVs did so by 30.04 ± 7.47% (*p* < 0.0001, *n* = 4), *E. faecalis*-derived EVs did so by 34.20 ± 11.65% (*p* < 0.0001, *n* = 6), and *G. vaginalis*-derived EVs did so by 37.01 ± 10.26% (*p* < 0.0001, *n* = 5).

### Cell Pre-Exposure With Bacterial EVs Does Not Prevent HIV-1 Infection

**Figure 6:**
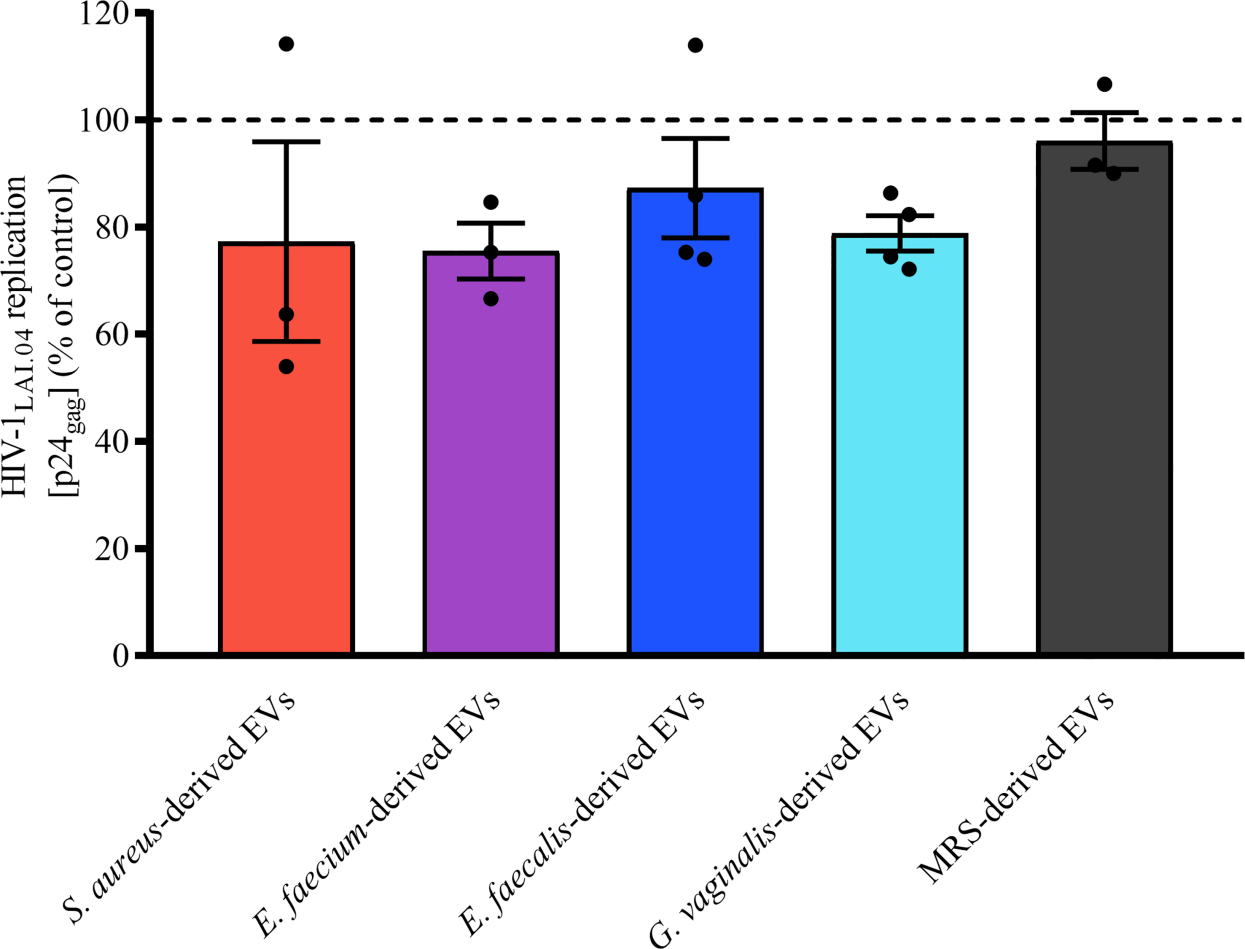
Effect of cell pre-exposure with bacterial EVs on HIV-1 infection. MT-4 cells were preincubated with bacterial EVs for 24 h, washed off, infected with HIV-1_LAI.04_, and incubated for 3 days. HIV-1 replication was evaluated from measurements of the capsid protein p24_gag_ in cell culture medium and is represented as a percentage of HIV-1 replication in untreated control. Presented are means ± SEM from at least three independent measurements.

To understand whether bacterial EVs inhibit HIV replication directly or indirectly by inducing host cell responses, we pre-incubated MT-4 cells with bacterial EVs for 24 h, washed off the EVs, and infected MT-4 cells with HIV-1_LAI.04_. As shown in **Figure 6**, there was no statistically significant decreasing effect on HIV-1_LAI.04_ infection when cells were pre-treated with 1x10^10^ bacterial EVs derived from *S. aureus* (*p* = 0.2516, *n* = 3), *E. faecium* (*p* = 0.1968, *n* = 3), *E. faecalis* (*p* = 0.6834, *n* = 4), or *G. vaginalis* (*p* = 0.2468, *n* = 4), or with particles derived from MRS medium (*p* = 0.9970, *n* = 3).

### Bacterial EVs Prevent HIV-1 Infection Affecting Viral Env, gp120

**Figure 7:**
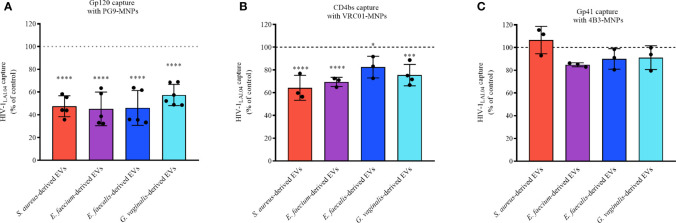
HIV-1 capture. HIV-1_LAI.04_ was pre-treated with 1×10^10^ EVs derived from *S. aureus*, *E. faecium*, *E. faecalis*, or *G. vaginalis*, or with particles derived from MRS medium, or with PBS (control) for 1 h. Next, HIV-1_LAI.04_ virions were captured with PG9 **(A)**, VRC01 **(B)**, or 4B3 **(C)** antibodies coupled to magnetic nanoparticles (MNPs). PG9 antibody recognizes HIV-1 trimeric gp120 proteins, VRC01 recognizes the CD4 binding site on the viral gp120, and 4B3 antibodies recognize the viral gp41. Data are presented as percentage of p24_gag_ concentration compared with the control. Presented are means ± SEM from three independent measurements. Asterisks indicate statistical significance by one-way ANOVA multiple comparison with Dunnett’s correction (*p < 0.05, ***p < 0.001, ****p < 0.0001).

To investigate whether bacterial EVs derived from *S. aureus*, *E. faecium*, *E. faecalis*, and *G. vaginalis* directly affect the HIV-1 viral envelope, in particular gp120 or gp41, we incubated 1×10^10^ bacterial EVs for 1 h with HIV-1_LAI.04_. Then, virions were captured using antibodies conjugated to magnetic nanoparticles (MNPs). We used PG9-MNPs, which recognize preferentially the HIV-1 trimeric envelope proteins gp120. VRC01-MNPs recognize the CD4 binding site (CD4bs) on the gp120 subunit. 4B3-MNPs recognize the viral gp41 subunit. Our results show that the incubation of HIV-1_LAI.04_ with bacterial EVs led to a significant reduction in the number of HIV-1 virions captured with PG9-MNP antibodies in comparison with the control (free of bacterial EVs) (**Figure 7A**). The numbers of virions captured decreased by 52.56 ± 9.23% (*p* < 0.0001, *n* = 5) when incubated with *S. aureus*-derived EVs, by 54.83 ± 14.78% (*p* < 0.0001, *n* = 5) when incubated with *E. faecium*-derived EVs, by 54.01 ± 15.33% (*p* < 0.0001, *n* = 5) when incubated with *E. faecalis*-derived EVs, and by 42.64 ± 9.32% (*p* < 0.0001, *n* = 6) when incubated with *G. vaginalis*-derived EVs (**Figure 7A**).

Similar results were obtained using VRC01 as capture antibody. The numbers of virions captured upon treatment with bacterial EVs were reduced by 35.67 ± 10.95% (*S. aureus*-derived EVs, *p* < 0.0001, *n* = 3), 30.52 ± 4.03% (*S. faecium*-derived EVs, *p* < 0.0001, *n* = 3), 30.52 ± 4.03% (*S. faecalis*-derived EVs, *p* = 0.0262, *n* = 3), and by 24.46 ± 9.40% (*G. vaginalis*-derived EVs, *p* = 0.0002, *n* = 4), compared with the EV-free control (**Figure 7B**).

No statistically significant reduction in the number of captured viruses was observed with 4B3-MNPs when treated with EVs derived from *S. aureus* (*p* = 0.9082, *n* =3), *E. faecium*-derived EVs (*p* = 0.2405, *n* = 3), *E. faecalis*-derived EVs (*p* = 0.6281, *n* = 3), or *G. vaginalis*-derived EVs (*p* = 0.7270, *n* = 3) (**Figure 7C**).

### The Protein Component of Pathogen EV Is Essential for HIV-1 Inhibition

Since our results suggest that bacterial-derived EVs inhibit HIV-1 infection by interacting with viral gp120, we wondered whether proteins exposed on the EV surface are responsible for this interaction. Toward this goal, EVs derived from *S. aureus*, *E. faecium*, *E. faecalis*, and *G. vaginalis* were treated with PK to digest any proteins on the EV surface.

**Figure 8:**
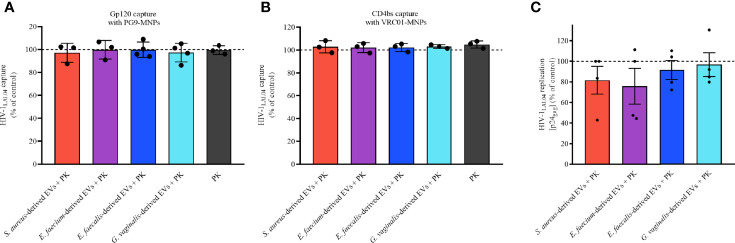
Bacterial EV-associated protein role during HIV-1 infection. The proteins associated to bacterial EVs were digested with PK followed by an ulterior ultracentrifugation to obtain bacterial EVs free of surface proteins. HIV-1_LAI.04_ was pre-treated with 1×10^10^ PK-treated EVs derived from *S. aureus*, *E. faecium*, *E. faecalis*, or *G. vaginalis*, or with PK (PBS treated with PK and purified by ultracentrifugation), or with PBS (control) for 1 h. Next, HIV-1_LAI.04_ virions were captured with PG9 **(A)** or VRC01 **(B)** antibodies. Also, the antiviral effect of PK-treated bacterial EVs in MT-4 cells infected with HIV_LAI.04_ was evaluated **(C)**. The amount of virus captured and the amount of virus present on cell culture medium were determined from measurement of the levels of viral p24_gag_. Data are presented as percentages of p24_gag_ concentration compared with the control (PBS). Presented are means ± SEM from three independent measurements. Statistical analysis was performed with one-way ANOVA multiple comparison.

As shown in **Figures 8A, B**, upon treatment with PK the capacities of bacterial-derived EVs to interact directly with viral gp120 were lost, as the number of virions captured with PG9-MNPs (**Figure 8A**) and VRC01-MNPs (**Figure 8B**) were similar to that in the EV-free control (100%). Indeed, capturing virus with PG9-MNPs (**Figure 8B**), we found no statistically significant effect on HIV-1_LAI.04_ envelope when virions were treated with PK-treated EVs derived from *S. aureus* (*p* = 0.9994, *n* = 3), *E. faecium* (*p* > 0.9999, *n* = 3), *E. faecalis* (*p* = 0.9994, *n* = 4), or *G. vaginalis* (*p* > 0.9999, *n* = 4), or with PK (*p* > 0.9999, *n* = 3). Similarly, capturing virus with VRC01-MNPs (**Figure 8B**), no statistically significant effect on HIV-1_LAI.04_ envelope was observed when virions were treated with PK-pretreated EVs derived from *S. aureus* (*p* = 0.9967, *n* = 3), *E. faecium* (*p* > 0.9994, *n* = 3), *E. faecalis* (*p* = 0.9999, *n* =2), or *G. vaginalis* (*p* = 0.9937, *n* = 3), or with PK (*p* > 0.9967, *n* = 3).

We also tested the anti-HIV-1 activities of bacterial EVs treated with PK in MT-4 cells. As shown in **Figure 8C**, the anti-HIV-1 activities of all bacterial EVs tested were completely lost after proteinase K treatment. Indeed, our results demonstrated that HIV-1_LAI.04_ replication was not reduced significantly in the presence of PK-treated bacterial EVs derived from *S. aureus* (*p* = 0.9654, *n* = 4), *E. faecium* (*p* = 0.8498, *n* = 4), *E. faecalis* (*p* = 0.9994, *n* = 4), or *G. vaginalis* (*p* = 0.9998, *n* = 4), compared with the control experiments (**Figure 8C**).

These results suggest that proteins exposed on bacterial EV surface are involved in the antiviral effect by obstructing the trimeric region and the CD4 binding site of viral gp120 protein.

### Proteins Associated With Bacterial EVs

**Figure 9:**
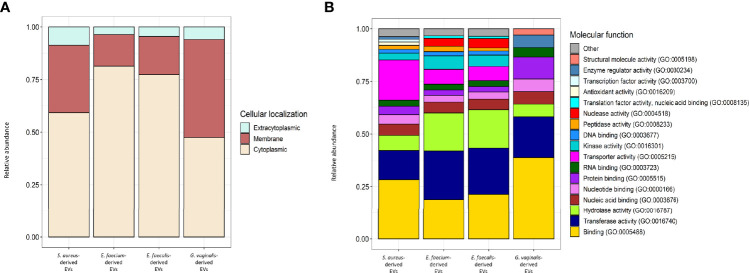
Cellular localization and molecular function GOs of *S. aureus*, *E. faecium*, *E. faecalis* and *G.vaginalis-*derived EV proteome. **(A)** Relative abundance of the proteins predicted to have a cytoplasmic, extracytoplasmic, or intracellular localization within each strain-derived EV proteome. Detailed results of TMHMM and SignalP analysis used to predict TM and signal peptide motifs in the EV-related proteins are reported in **Tables S1–S4**. **(B)** Relative abundance of the proteins belonging to each molecular function’s GO within each strain-derived EV proteome; ‘other’ includes GO terms with a relative abundance < 1%. Molecular functions were extracted from strain-derived EV proteomes, analyzed in topGO, and categorized in CateGOrizer (Hu et al., 2008) against the GOSlim2 database. Raw data are reported in **Tables S1–S11**; sa and gv indicate molecular functions that were identified only in the *S. aureus* and *G. vaginalis-*derived EVs proteome, respectively.

Using liquid chromatography–mass spectroscopy, we investigated the proteins associated to bacterial EVs. We identified 390, 960, 1,158, and 118 bacterial proteins associated to EVs derived from *S. aureus, E. faecium, E. faecalis*, and *G. vaginalis*, respectively (**Table S1**). In terms of cellular localization by gene ontology (GO) terms, more than 90% of the EV-associated proteins were predicted to have cytoplasmatic and trans-membrane localizations, with less than 10% of them being extracytoplasmic (**Figure 9A**). According to the molecular function by the GO terms, EVs derived from *S. aureus, E. faecium*, and *E. faecalis* shared most of their molecular functions. However, the EV-derived proteome of *G. vaginalis* showed some differences from the other strains. In this regard, EV-derived proteins with binding activity, transferase activity, transporter activity, hydrolase activity, and nucleotide/nucleic acid binding activity were shared among all the strains under analysis (**Figure 9B**), while an additional five molecular functions (protein and DNA binding, kinase and peptidase activity, and transporter activity) were shared only among *S. aureus*, *E. faecium* and *E. faecalis*. (**Figure 9B**). On the other hand, proteins with structural molecule activity and translation factor activity were detected only in the *G. vaginalis* EV-related proteome (**Figure 9B**).

**Figure 10:**
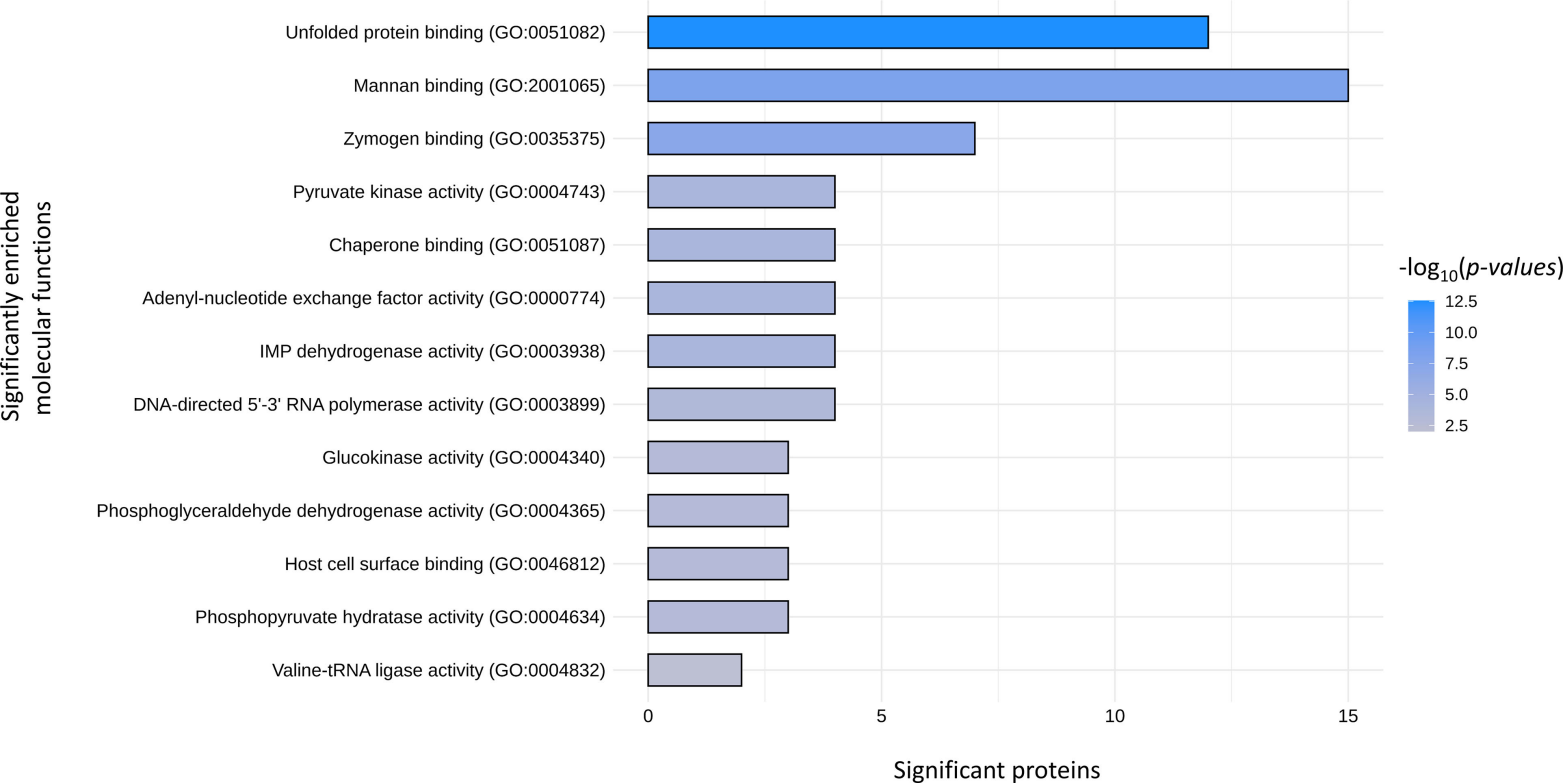
Molecular function GOs significantly enriched in the EV derived proteins that are shared among *S. aureus*, *E. faecium*, *E. faecalis* and *G.vaginalis*. Raw data used for the plot are reported in **Tables S1–S11**.

Comparative analysis of EV-derived proteomes identified a total of 30 clusters of orthologue (OG) proteins shared among *S. aureus*, *E. faecium*, *E. faecalis* and *G. vaginalis* strains (**Table 1**). These clusters included proteins mostly involved in basal cellular functions such as translation (OG25, OG50, OG49, OG55, OG15 and OG30), transcription (OG51, OG56, OG69, OG68), chaperones and chaperonins (OG66, OG60 OG54, OG52), components of the ABC transporters (OG00 and OG28), and enzymes of the glycolytic pathways (OG53, OG48, OG11 and OG09). Significantly enriched GOs were identified from comparisons of the molecular functions of the EV-derived proteins included in the shared OG clusters with the whole EV-derived proteome from each strain. As a result, significantly enriched GO terms within the shared proteins corresponded to molecular functions involved in the binding of unfolded proteins (GO:0051082), nucleoside (GO:0003938) and nucleotide (GO:0000774) catalytic activity, interaction with the host cells (GO:2001065; GO:0035375), and catalytic activities of the glycolytic pathway (GO:0004743; GO:0004340; GO:0004365; GO:0004634) (**Figure 10**).

**Table 1:** Description of EV-derived proteins included in orthogroups (OG) shared between *S. aureus*, *E. faecium*, *E. faecalis*, and *G. vaginalis*.

*Shared OG^a^*	Description
*OG69*	DNA-directed RNA polymerase subunit beta’
*OG68*	DNA-directed RNA polymerase subunit beta
*OG67*	Acetate kinase
*OG66*	Protein GrpE
*OG64*	ATP synthase gamma chain
*OG63*	Ferritin-like protein
*OG62*	ATP synthase subunit beta
*OG60*	Chaperone protein DnaK
*OG58*	60 kDa chaperonin
*OG56*	DNA-directed RNA polymerase subunit alpha
*OG55*	50S ribosomal protein L4
*OG54*	10 kDa chaperonin
*OG53*	Pyruvate kinase
*OG52*	Trigger factor
*OG51*	Elongation factor G
*OG50*	50S ribosomal protein L10
*OG49*	50S ribosomal protein L21
*OG48*	Enolase
*OG47*	Glucokinase
*OG46*	30S ribosomal protein S10
*OG30*	Serine-tRNA ligase
*OG28*	ABC transporter, substrate-binding protein^b^
*OG25*	Elongation factor Tu
*OG24*	Glutamine synthetase
*OG15*	Isoleucine-tRNA ligase
*OG11*	NAD-dependent glyceraldehyde-3-phosphate dehydrogenase
*OG10*	Lipoprotein^b^
*OG09*	L-lactate dehydrogenase
*OG08*	Inosine-5’-monophosphate
*OG00*	ABC transporter ATP-binding protein^b^

a The full list of proteins from each strain included in OG is reported in **Table S5**.

b For OGs including heterogeneous names, the protein family name was used.

## Discussion

Based on the establishment of complex interactions, the microbial communities that populate the human body are well-balanced and perfectly distributed with the host, other microorganisms, and the environment. The variations of a single bacterial component may perturb the entire ecosystem and lead to a deep rearrangement in the community composition, with possible onset of different diseases.

*Lactobacillus* species in vaginas of healthy pre-menopausal women represent the first barrier against numerous urogenital pathogens. Indeed, it has been shown that several *Lactobacillus* strains, such as *L. crispatus*, *L. gasseri*, and *L. jensenii*, reduce the possibility of acquisition of sexually transmitted infections, such as HIV-1 (Petrova et al., 2013). We reported that vaginal lactobacilli exert anti-HIV-1 activity by multiple mechanisms, including lactic acid production, acidification of the niche, and more recently by the release of nano-sized EVs (Ñahui Palomino et al., 2017; Ñahui Palomino et al., 2019).

Although *Lactobacillus* dominates in healthy vaginas, other bacteria that are considered pathogenic, such as *Gardnerella* spp., *Enterococcus*, *Staphylococcus*, etc., are present as well but are kept under control (Petrova et al., 2013; Gosmann et al., 2017). Here, we found that some of these bacteria secrete vesicles that confer protection against HIV-1 transmission.

In the last decade, the number of studies on bacterial EVs has been rapidly evolving, as demonstrated by their implication in bacteria–bacteria and bacteria–host interactions, either promoting health or causing various pathologies (Ñahui Palomino et al., 2021).

Specifically, we investigated whether three strains of Gram-positive bacteria (*S. aureus*, *E. faecium*, and *E. faecalis*) and the Gram-variable bacteria *G. vaginalis*, which can be present in the vaginas of healthy women (Ravel et al., 2011), release EVs capable of counteracting HIV-1 infection. We found that *S. aureus*, *E. faecium*, *E. faecalis*, and *G. vaginalis* release EVs similar in size (~ 200 nm) and comparable to those previously reported for other bacteria (Askarian et al., 2018; Wagner et al., 2018a; Kim et al., 2019; Shishpal et al., 2020).

We investigated whether, as with several *Lactobacillus* strains (Ñahui Palomino et al., 2019), EVs released by *S. aureus*, *E. faecium*, *E. faecalis*, and *G. vaginalis* may contribute to a protective effect against HIV-1 infection. Towards this goal, we tested the anti-HIV-1 effect of bacterial EVs in immortalized human lymphoid cells and in human cervico-vaginal tissues *ex vivo*. *Ex vivo* human tissues faithfully reflect many aspects of the tissues *in vivo*, where critical events of HIV-1 pathogenesis and transmission occur. In particular, they retain the key cell-surface molecules to support HIV-1 infection. Tissues *ex vivo* have been used widely to study host–pathogen interaction during HIV-1 infection (Grivel and Margolis, 2009; Ñahui Palomino et al., 2017; Ñahui Palomino et al., 2019).

Our results demonstrate that all tested bacteria release antiviral factors, as their cell supernatants inhibited HIV-1 replication in human T cells. The strength of this inhibition was comparable with that reported earlier for the supernatant of vaginal lactobacilli (*L. crispatus* BC3, *L. crispatus* BC5, *L. gasseri* BC12, and *L. gasseri* BC13) (Ñahui Palomino et al., 2017).

Next, we checked whether EVs that are present in the supernatants from the bacterial cultures can protect human cervico-vaginal tissues *ex vivo* as well as T-cell line from HIV-1 infection. Indeed, we found that EVs isolated from *S. aureus*, *E. faecium*, *E. faecalis*, and *G. vaginalis* largely protected human cervico-vaginal tissues *ex vivo* as well as a T-cell line from HIV-1 infection. The protection was observed using two HIV-1 strains which recognise different co-receptors for cell entry (CXCR4 for HIV-1_LAI.04_ and CCR5 for HIV-1_BaL_), suggesting a common and co-receptor–independent antiviral effect exerted by bacterial EVs. The anti-HIV-1 effect of EVs was concentration-dependent, and the inhibition of HIV-1 replication was not due to EV-induced cell death. Moreover, these anti-HIV-1 activities were comparable to the anti-HIV-1 activity exerted by other Gram-positive bacteria–derived EVs (*L. crispatus* BC3-derived EVs, *L. gasseri* BC12-derived EVs) in human T-cell lines and human tissues *ex vivo* (tonsillar and cervico-vaginal tissues) (Ñahui Palomino et al., 2019).

In principle, EVs may directly affect the infectivity of HIV-1 virions or may indirectly inhibit HIV-1 infection by interacting with viral target cells, or both. Pre-incubation of bacterial EVs with cells, at least for 24 h, did not result in HIV-1 inhibition. Therefore, we did not investigate further the bacterial EV-host cell interactions. In contrast, we found that the reduction of HIV-1 replication can be related to the alteration of HIV-1 virions by all bacterial-derived EVs tested.

One meaningful strategy to prevent HIV-1 infection is to target the viral gp120, which facilitates viral entry into the target cell. Steric hindrance or blocking the gp120 trimeric region and CD4 binding site on HIV-1 envelope is a key aspect of such strategy. Accordingly, numerous broadly neutralizing antibodies, nanobodies, or small molecules targeting viral gp120 have been developed. A small molecule that directly interacts with gp120 protein, Fostemsavir, was recently approved for medical treatment in the United States (2020) and in Europe (2021).

It seems that *S. aureus*, *E. faecium*, *E. faecalis*, and *G. vaginalis* use a similar anti-HIV-1 strategy as virions exposed to EVs released by these bacteria were no longer being recognized by MNP-coupled PG9 antibodies, which bind to the functional trimeric form of viral gp120. Similar results were observed when VRC01 antibodies, which recognize the CD4bs on the gp120, were used. These observations indicate that bacterial EVs interact specifically with the gp120 subunit of the HIV-1 envelope, as the virus treated with bacterial EVs and its subsequently capture with 4B3 antibodies, which recognize gp41, did not alter the amount of virus captured. Altogether, our results obtained in the present study are comparable with those previously reported for *Lactobacillus*-derived EVs (Ñahui Palomino et al., 2019), suggesting that HIV-1 inhibition by steric hindrance of gp120 or gp120 modification is a common mechanism of Gram-positive bacteria.

Here, we showed that proteins associated to bacterial EVs are important for this anti-HIV-1 effect. Toward this goal, we treated bacterial EVs with proteinase K (PK). PK-treated EVs lost their ability to inhibit HIV-1 replication in human T cells, suggesting that proteins exposed on the external surface of EVs are involved in the EV-mediated anti-HIV-1 mechanism. In agreement with these results, PK-treated EVs did not alter viral capture by PG9 or VRC01 antibodies, compared with controls.

We identified proteins associated to bacterial EVs that may be responsible for their anti-HIV-1 effect. Among the proteins detected in all the bacterial EVs, 30 orthogroup proteins (clusters of orthologue proteins) were common to the EV-proteome of all four strains. Similar EV-associated proteins were found in EVs released by *S. aureus*, *E. faecalis*, and *G. vaginalis*, as reported by others (Wagner et al., 2018b; Shishpal et al., 2020; Tartaglia et al., 2020). None of these bacterial 30 orthogroup proteins were previously reported to interact with HIV-1 or other viruses. However, human homologues of these proteins, such as chaperone protein DnaK (Hsp70), enolase (ENO), elongation factor TU (EFTU), and glyceraldehyde-3-phosphate dehydrogenase (GAPDH), are involved during HIV-1 infection (Cimarelli and Luban, 1999; Kumar et al., 2011; Kishimoto et al., 2012; Li et al., 2015; Kishimoto et al., 2020). In particular, Hsp70 decreases HIV-1 replication by inhibiting viral gene expression (Kumar et al., 2011). Likewise, *Mycobacterium* Hsp70 has been shown to interact with CCR5, abrogating HIV-1 infection of human CD4^+^ T cells (Babaahmady et al., 2007). Overexpression of ENO in HIV-1 target cells decreases HIV-1 infection by inhibiting viral integration and replication (Kishimoto et al., 2020). EF1α (eukaryotic homologue of EFTU) and GAPDH oppositely impact the reverse transcription of viral RNA: EF1α positively influences the reverse transcription by interacting with 5’UTR of HIV-1, while GAPDH negatively regulates the reverse transcription of viral RNA (Kishimoto et al., 2012; Li et al., 2015). Also, EF1α interacts with HIV-1 gag proteins and can be incorporated in the HIV-1 virion (Cimarelli and Luban, 1999). Furthermore, in EVs with anti-HIV-1 activity, such as EVs derived from *L. gasseri*, ENO-2, EFTU, ATP synthase gamma chain, and 60 kDa chaperonin (Ñahui Palomino et al., 2019; Costantini et al., 2021) have also been found. Whether HIV-1 inhibition by bacterial EVs is directly or indirectly exerted by one or more of these proteins remain to be elucidated.

It has been reported that changes in microbial composition in the vaginal environment from *Lactobacillus* dominance to non-*Lactobacillus* species such as *G. vaginalis* increase the susceptibility to HIV-1 infection (Sha et al., 2005; Ravel et al., 2011; Klatt et al., 2017). However, the exact mechanisms of this phenomenon still need to be fully elucidated. The ability of each bacterium to increase or decrease the susceptibility to HIV-1 infection will depend on the bacteria itself, release of virulence factors, capabilities to disrupt the host epithelial barrier, their capacities to induce pro-inflammatory environment that attract HIV-1 target cells, etc (Petrova et al., 2013). The final effect of vaginal microbiota on HIV-1 infection may be a balance of all the effects. Here, we focused on the study of EVs produced by *S. aureus*, *E. faecium*, *E. faecalis*, and *G. vaginalis*. If our results are extrapolated to *in vivo* HIV-1 transmission, it would be important to determine the amount of EVs produced by bacteria *in vivo*. A limiting factor for the comprehension of the real impact exerted by EVs is a lack of specific tools for the isolation of bacterial EVs released *in vivo*. Identification of antiviral molecules present in EVs, as well as their mechanism of action, will be fundamental for understanding HIV-1 protection mediated by bacterial EVs *in vivo*.

Although antiviral properties have never been reported before for *S. aureus*, *E. faecalis*, and *G. vaginalis*, *E. faecium* has been shown to exert antiviral activity against transmissible gastroenteritis coronavirus (TGEV), swine influenza A, astrovirus, and rotavirus A in *in vivo* and in *in vitro* studies (Kreuzer et al., 2012; Chai et al., 2013; Wang et al., 2013). Also, *E. faecium*, induced the upregulation of proinflammatory cytokines (IL-6, IL8) and nitric oxide on epithelial swine testicle cells, with a consequent reduction of viral infectivity. Moreover, *E. faecium* was able to trap TGEV virions, as observed with electron microscopy, decreasing viral infectivity (Kreuzer et al., 2012; Chai et al., 2013; Wang et al., 2013). Now, it should be investigated whether EVs generated by these bacteria mediate these activities.

In conclusion, EVs released by Gram-positive bacteria (*S. aureus*, *E. faecium*, *E. faecalis*) and *G. vaginalis*, protect human cervicovaginal tissues *ex vivo* as well as isolated human cells from HIV-1 infection. This protection is not due to a cytotoxic effect of bacterial EVs but rather to steric hindrance of gp120 or gp120 modifications. EVs carry numerous bacterial proteins which may be associated with the anti-HIV-1 effect. Identification of the bacterial EV molecules responsible for this anti-HIV-1 effect may help to the development of new therapeutic agents that prevent HIV-1 vaginal transmission.

## Data Availability Statement

The datasets regarding relative abundance of proteins identified in bacterial EVs and presented in the study are reported in the **Supplementary Material** and are also available upon a request to the authors.

## Author Contributions

PC and RÑ designed and performed the experiments, analyzed the data, and wrote the manuscript. LM and CV designed experiments, analyzed the data, and wrote the manuscript. AF and MC analyzed the proteomic data. All the authors contributed to data interpretation. All the authors read, reviewed, and approved the final manuscript.

## Funding

This work was supported by the NICHD Intramural Program.

## Conflict of Interest

The authors declare that the research was conducted in the absence of any commercial or financial relationships that could be construed as a potential conflict of interest.

## Publisher’s Note

All claims expressed in this article are solely those of the authors and do not necessarily represent those of their affiliated organizations, or those of the publisher, the editors and the reviewers. Any product that may be evaluated in this article, or claim that may be made by its manufacturer, is not guaranteed or endorsed by the publisher.

## Supplementary Material

Supplementary Table 1-11 Functional annotation and cellular organization of EVs-related proteins quantified through proteomic analysis in *S. aureus*, *E. faecium*, *E. faecalis* and *G. vaginalis*.
